# CNN-Based Person Detection Using Infrared Images for Night-Time Intrusion Warning Systems

**DOI:** 10.3390/s20010034

**Published:** 2019-12-19

**Authors:** Jisoo Park, Jingdao Chen, Yong K. Cho, Dae Y. Kang, Byung J. Son

**Affiliations:** 1 School of Civil and Environmental Engineering, Georgia Institute of Technology, Atlanta, GA 30332, USA; jpark711@gatech.edu (J.P.); jchen490@gatech.edu (J.C.); yong.cho@ce.gatech.edu (Y.K.C.); 2 College of Sports and Leisure Studies, Yonsei University, Seoul 03722, Korea; ctokang@lskorea.org; 3 Department of International Civil & Plant Engineering, Konyang University, Nonsan 32992, Korea

**Keywords:** lifesaving, human detection, deep learning, CNN, infrared image

## Abstract

Night-time surveillance is important for safety and security purposes. For this reason, several studies have attempted to automatically detect people intruding into restricted areas by using infrared cameras. However, detecting people from infrared CCTV (closed-circuit television) is challenging because they are usually installed in overhead locations and people only occupy small regions in the resulting image. Therefore, this study proposes an accurate and efficient method for detecting people in infrared CCTV images during the night-time. For this purpose, three different infrared image datasets were constructed; two obtained from an infrared CCTV installed on a public beach and another obtained from a forward looking infrared (FLIR) camera installed on a pedestrian bridge. Moreover, a convolution neural network (CNN)-based pixel-wise classifier for fine-grained person detection was implemented. The detection performance of the proposed method was compared against five conventional detection methods. The results demonstrate that the proposed CNN-based human detection approach outperforms conventional detection approaches in all datasets. Especially, the proposed method maintained F1 scores of above 80% in object-level detection for all datasets. By improving the performance of human detection from infrared images, we expect that this research will contribute to the safety and security of public areas during night-time.

## 1. Introduction

Night-time intrusion warning systems are some of the most important applications of IoT (Internet of Things) technology. Night-time intruders into dangerous areas face a high risk of injury or death due to the lack of safety personnel during the night and limited visibility. Having an automated warning system to deter intruders is crucial for both the safety of the general public and the security of restricted areas. One of the most important applications for night-time intrusion warning is drowning prevention. According to the World Health Organization (WHO), 372,000 people die due to drowning accidents every year [1]. Although most of the drowning accidents happen during daytime, the night-time intrusion warning system for drowning prevention is important because during the night-time, lifeguards might be absent, and rescue operations are more difficult [2]. As part of ongoing efforts to prevent these drowning accidents, several intelligent surveillance systems have been developed using closed-circuit television (CCTV) cameras paired with computer vision and machine learning technologies [2,3,4]. These systems automatically detect people swimming between the established boundaries in CCTV images and issue an alert to the supervisor whenever the system finds someone trespassing across the safety lines [4]. However, most systems operate with images taken in daylight, whereas the performance for detecting people in the water during the night-time is considerably worse.

To detect people more effectively at night, several studies have adopted machine learning-based approaches using specific features extracted from infrared camera images. These machine learning-based human detection approaches can be classified according to the type of feature descriptors and classifiers that are applied. For example, histograms of oriented gradients (HOG) [5,6], scale-invariant feature transform (SIFT)-like oriented features [7], and intensity distribution-based inertia features (INERTIA) [8] are widely used for feature extraction, and support vector machines (SVM) [9,10,11], or AdaBoost [11] are frequently used as classifiers. In addition, the Haar-Cascade classifier, which is a well-known object detection method based on Haar-like features with boosted cascade classifiers, is also adopted for human detection in infrared images in several studies [12,13,14]. If proper features and feature extraction techniques are selected, classical machine learning approaches usually show satisfactory detection performance. On the other hand, these approaches suffer from low detection performance in images containing ambiguous or noisy features [15,16,17].

To address this problem, deep learning approaches based on convolution neural networks (CNN) have recently been presented for person detection in infrared images [18,19,20,21]. These deep learning-based approaches can enhance the object detection performance in infrared images since the increased model complexity leads to better learning capacity. It has been found in the literature that CNN-based human detection approaches show better detection rates compared to SVM-based classification approaches [16,22]. The detection performance of deep learning-based approaches depends on having the right training data; thus, constructing an appropriate dataset for the specific situation of detecting swimmers at night-time is critical. Several studies have developed infrared image datasets such as KAIST [23], CVC-14 [24], and LSI [25] for human detection during night-time. However, since these infrared image datasets aim to detect pedestrians, they consist of images captured from cameras installed on a ground vehicle. The people in these images have a significantly different appearance compared to that of CCTV installed on the beach for safety purposes in which people are represented by only a few pixels (i.e., part of the body above water). Therefore, in order to achieve the purpose of detecting people in CCTV images for long-range surveillance during night-time, there is a need for more specific datasets and a more fine-grained object detection method. 

This research proposes an accurate and efficient method for detecting people in infrared CCTV installed at overhead locations during the night-time. For this purpose, a CNN-based pixel-wise classifier for fine-grained person detection was proposed. This study acquired three different datasets based on images taken with infrared cameras from an overhead point-of-view. In addition, this paper also proposes a modified CNN model in which the three input channels are taken from an (i) original infrared image, (ii) difference image from the previous frame, (iii) background subtraction mask. To verify the proposed methods, a comparative analysis with four different human detection methods is conducted. The main contributions of this research are

(i) to introduce a fine-grained person detector that is 10–30% more accurate compared to baseline methods;
(ii) to demonstrate the ability to perform long-range object detection under challenging conditions;
(iii) to validate the ability of the trained detector to generalize across different datasets.

## 2. Methodology

### 2.1. Composition of Datasets

**Figure 1:**
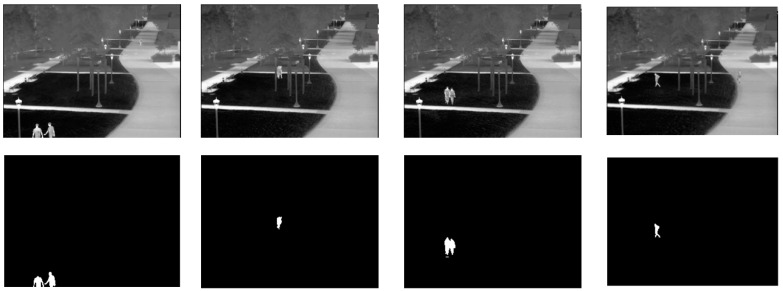
Example images and ground truth labels for the university dataset.

**Figure 2:**
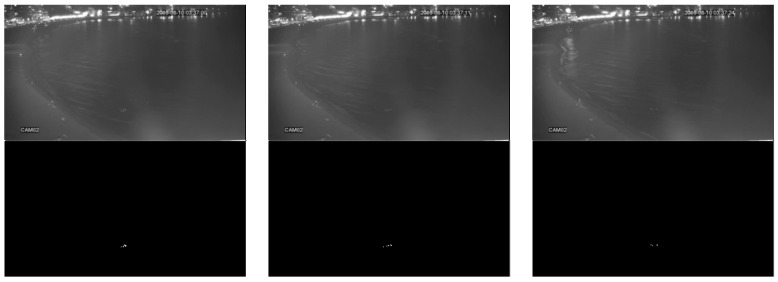
Example images and ground truth labels for the beach dataset.

**Figure 3:**
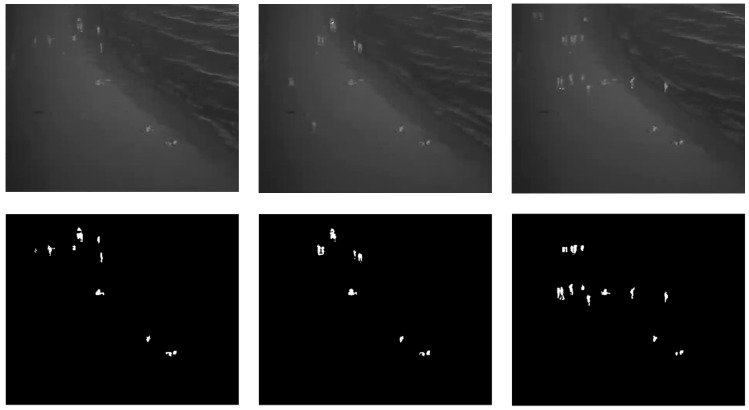
Example images and ground truth labels for the shore dataset.

This research used three infrared image datasets, named “university”, “beach”, and “shore”, for training and testing the person detection algorithms. The “university” dataset was taken from on top of a pedestrian bridge at a university campus using an infrared (FLIR) camera. The “beach” and “shore” datasets were taken from a rooftop of a building next to a public beach in Korea using an infrared CCTV. All datasets were collected at night-time and labeled at the pixel level. Table 1 shows the details of the datasets whereas Figure 1, Figure 2 and Figure 3 depict sample original and labeled images in all datasets. All datasets were split into 80% training data and 20% test data. The split was carried out while ensuring that the images in the training data and test data occur at different time segments in the original video.

**Figure 4:**
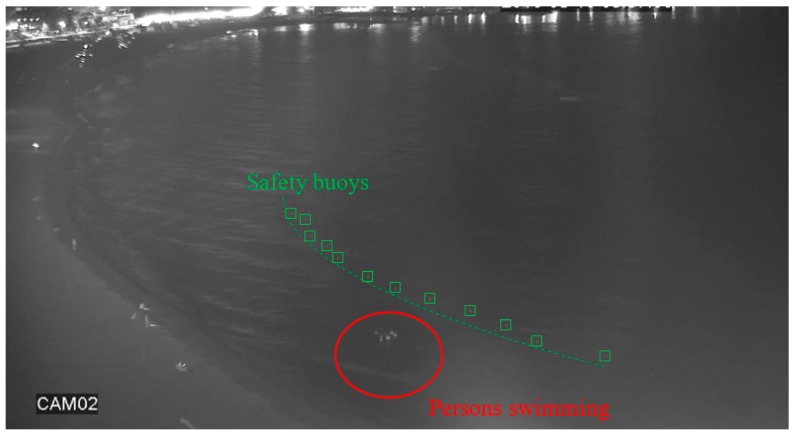
Visualization of the area of interest for surveillance in the beach dataset.

The “university” dataset was used to benchmark the performance of night-time human detection from an overhead viewpoint in brightly lit urban areas. The dataset was taken on a university campus with many different light sources that pose a challenge to intensity-based detection algorithms. On the other hand, the “beach” and “shore” datasets were used to practically evaluate human detection for night-time drowning prevention. Figure 4 shows the layout of the night-time scene captured by the beach CCTV camera. The scene consists of a public beach bordered by safety buoys with several groups of people scattered throughout. The task of interest was to automatically detect any persons swimming in the sea area, which is prohibited during night-time. This is a challenging task because the area of interest only constitutes a small region in the image since the camera is located far from the scene. Other challenging factors include noise from the waves and irregular lighting from the surrounding buildings. To partially overcome these problems, the original image was cropped to contain only the area of interest which is the sea area close to the beach. For this reason, only the people that intruded into the water area were detected, whereas the people on the beach were not detected.

### 2.2. Human Detection from Infrared Images

This research implemented six different methods of detecting people in infrared images including our proposed method and five baseline methods: (i) simple thresholding, (ii) adaptive thresholding, (iii) background subtraction, and (iv) K-means clustering and (v) Convolutional Neural Networks (CNN). The detection task was formulated as binary classification at the pixel level, i.e., each pixel is classified as either part of a person or not. This allows for a fine-grained detection where the exact object outline can be determined compared to bounding boxes which are inexact and only give a rough approximation of the object [26]. 

#### 2.2.1. Human Detection Using Conventional Computer Vision

The simplest method of detecting humans in infrared images is through thresholding. The technique of simple thresholding applies a constant threshold to the pixel intensity to determine pixels that correspond to humans. Since humans usually have a higher temperature than the surroundings, the corresponding pixels in infrared images will have higher intensities. Compared to simple thresholding, adaptive thresholding does not use a fixed threshold but a varying threshold that is determined by the Gaussian-weighted sum of the local intensity values. This allows the thresholding procedure to be more robust to changes in lighting conditions.

This study also considers the background subtraction method based on the fact that humans usually move relative to the surrounding environment. With background subtraction, the human can be distinguished from a sequence of infrared images as pixels that are changing over time in contrast to pixels that remain constant. This method is applicable in this research because the CCTV camera is installed with a fixed location and viewing angle; thus, any substantial change in pixel values can be attributed to moving objects. The background subtraction method implemented is based on the method of [27], where Gaussian mixture models are used to represent pixel intensity variation. 

In addition, the K-means clustering method, which is commonly used to segment regions of an image that have a certain range of intensity values [28,29,30], was considered. The intuition is that the foreground objects such as humans will have a certain distribution of pixel values that is distinct from that of background pixels. Thus, the K-means algorithm was used to determine the mean of each distribution (i.e., foreground vs. background) so that foreground and background pixels can be separated.

#### 2.2.2. Human Detection Using Deep Neural Networks

Deep neural networks have been shown in the literature to demonstrate state-of-the-art performance in various image processing tasks [31,32,33]. Even though infrared images may have a different appearance compared to visual images, similar techniques can still be applied. This study used CNN designed for the task of semantic image segmentation. The architecture has a base network of ResNet [31] and top layers with atrous convolutions [34]. The network takes in inputs of N×N×3 images and outputs N×N pixel-wise labels. The source code implemented in this paper has been made publicly available on GitHub (https://github.com/jingdao/IR_detection).

**Figure 5:**
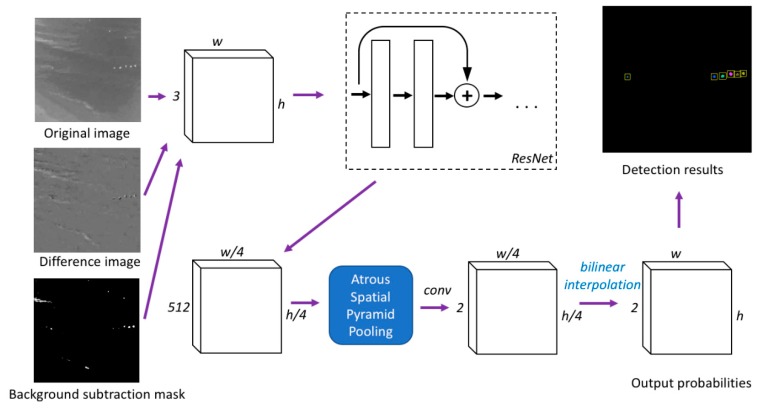
The network architecture of the proposed person detection method.

Figure 5 shows the detailed network architecture. The three input channels were taken from an (i) original infrared image, (ii) difference image from the previous frame, (iii) background subtraction mask. This is so that the network is able to capture temporal information as well when making predictions. The network was trained on the binary classification task using the cross-entropy loss. The output of the network is a 2D array of confidence scores for each pixel. The array is filtered with a detection threshold of 0.99 to create a binary mask. The high level of the detection threshold is used to ensure that noise in the detection results is adequately filtered out. To determine the actual number of persons, the connected component algorithm was used to merge neighboring pixels together in the binary mask to form individual objects. The network was trained for a total of 150 epochs with a learning rate of 0.001 and a batch size of 5.

**Figure 6:**
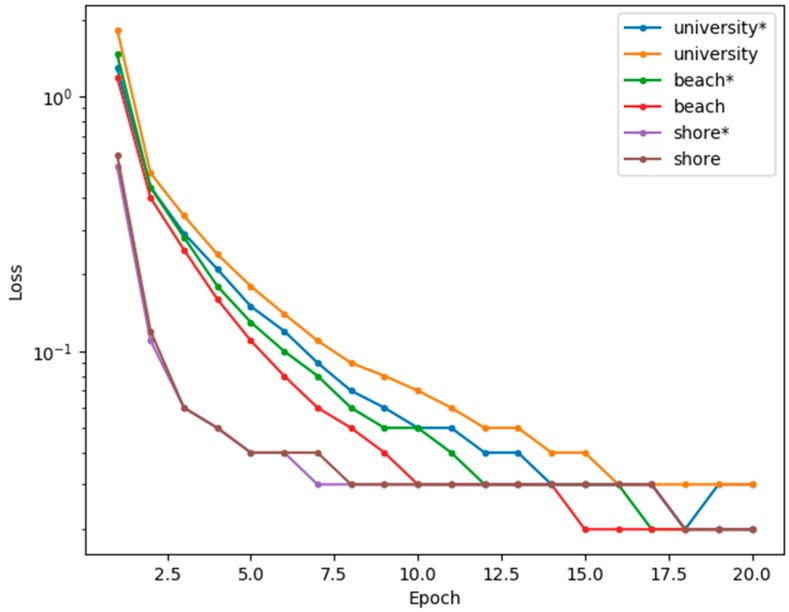
Loss function (logarithmic scale) of the first 20 epochs during training for each dataset. (*) indicates that temporal input is used.

Two network models were trained for each dataset. In the rest of this paper, “university*”, “beach*”, and “shore*” refer to the scenario where all three input channels (i.e., containing temporal information) were used whereas “university”, “beach”, and “shore” refer to the scenario where only the first input channel was used (i.e., no temporal information). Figure 6 shows a graph of the training loss for each dataset for the first 20 epochs of training. The training progress shows a similar trend for all datasets, where the loss function rapidly decreases during the first few epochs and then gradually plateaus afterward. The graph also shows that the training loss is generally higher for the “university” dataset compared to the “beach” and “shore” datasets. This may be because the “university” dataset has a wider range of pixel intensity values since it is in a lighted urban scene.

There are several challenges in implementing a segmentation network for our particular dataset compared to conventional visual images. First, the pixels that correspond to positive detections only occupy small regions in the image since the images are taken from an overhead view. To overcome this problem, a modified version of ResNet was used with a much smaller output stride while reducing the feature map depth to keep the memory consumption at a reasonable level. This allows the network to make fine-grained predictions in terms of the pixel neighborhood. Additionally, since the label distribution is severely skewed (each image has around 99% background pixels and only 1% foreground pixels), a weighted cross-entropy loss function was used where the loss from background pixels and foreground pixels were separately weighted based on their frequencies. 

## 3. Experimental Results

The performance of the proposed human detection method in infrared images was evaluated through the “university”, “beach”, and “shore” datasets. The evaluation metrics are (i) precision, (ii) recall, and (iii) F1-score, measured at both the pixel level and the object level. The proposed method was evaluated under two configurations: one where only the first input channel was used (i.e., no temporal information) and one where all three input channels (i.e., containing temporal information) were used (indicated by *). The proposed method was compared against five baseline methods which were (i) simple thresholding, (ii) adaptive thresholding, (iii) background subtraction, and (iv) K-means clustering (v) baseline CNN. The baseline CNN is a semantic segmentation CNN adapted directly from [34] without any of our modifications such as output stride tuning or weighted loss function.

**Figure 7:**
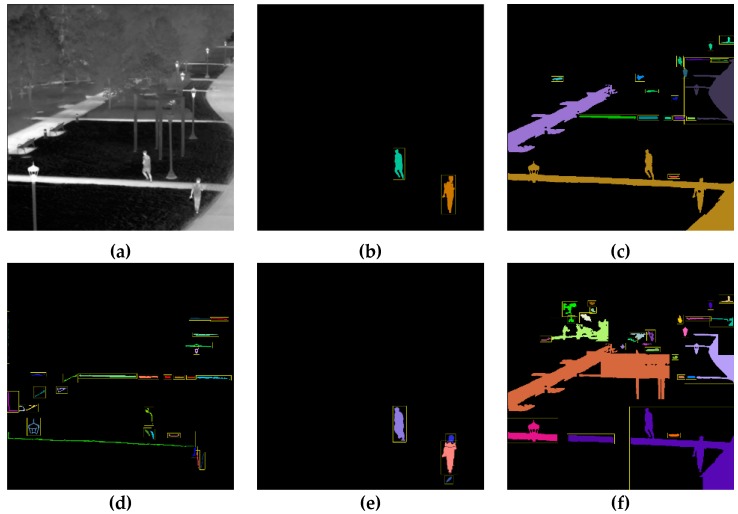

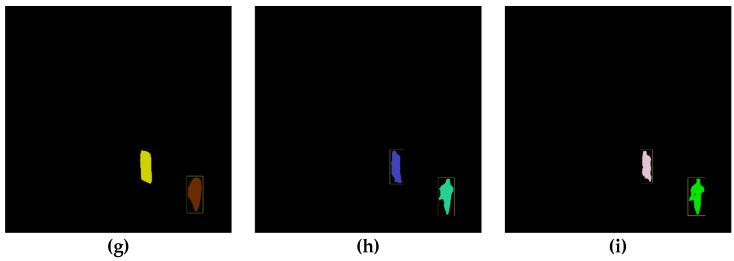
Detection results for the university dataset: (**a**) original infrared image, (**b**) ground truth, (**c**) simple thresholding, (**d**) adaptive thresholding, (**e**) background subtraction, (**f**) K-means clustering, (**g**) baseline convolution neural network (CNN), (**h**) our method, and (**i**) our method*.

**Figure 8:**
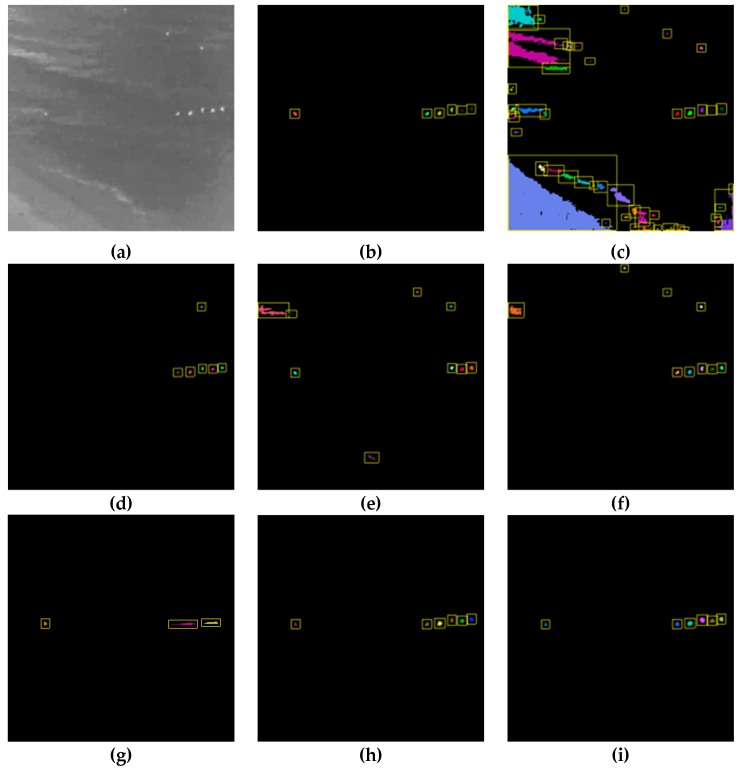
Detection results for the beach dataset: (**a**) original infrared image, (**b**) ground truth, (**c**) simple thresholding, (**d**) adaptive thresholding, (**e**) background subtraction, (**f**) K-means clustering, (**g**) baseline CNN, (**h**) our method, and (**i**) our method*.

**Figure 9:**
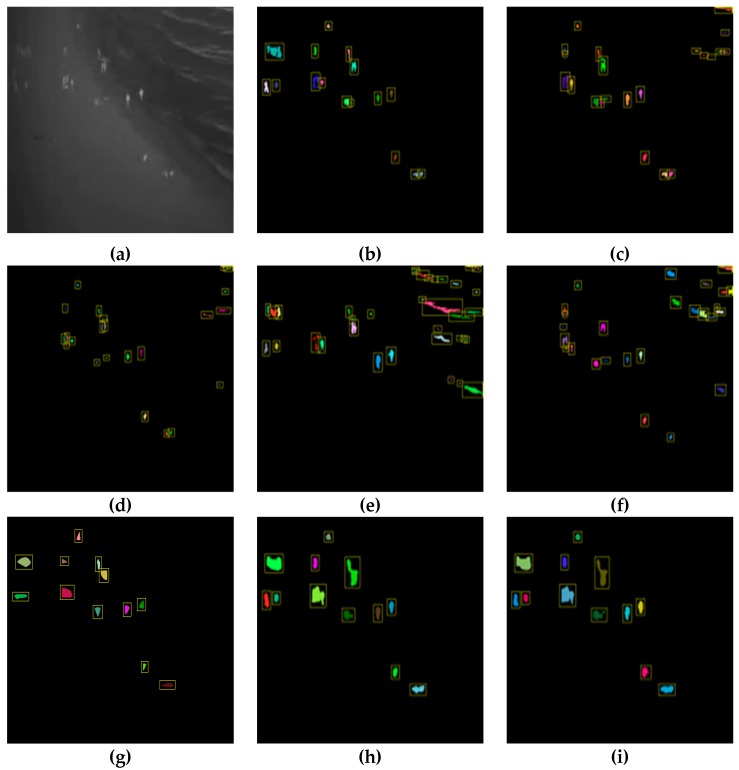
Detection results for the shore dataset: (**a**) original infrared image, (**b**) ground truth, (**c**) simple thresholding, (**d**) adaptive thresholding, (**e**) background subtraction, (**f**) K-means clustering, (**g**) baseline CNN, (**h**) our method, and (**i**) our method*.

Figure 7, Figure 8 and Figure 9 show a visualization of the detection results compared to the baseline methods whereas Table 2, Table 3, Table 4, Table 5, Table 6 and Table 7 show the numerical results. Figure 7, Figure 8 and Figure 9 show that our method obtains much better detection results, almost approaching the ground truth, compared to the baseline methods. Our method is able to correctly detect humans while ignoring background objects such as lamp posts and waves. This is confirmed by the numerical results in Table 2, Table 3, Table 4, Table 5, Table 6 and Table 7, where our method obtains the best pixel-level and object-level F1-scores in all datasets. In the “university” dataset, the use of temporal information as CNN input is able to improve the detection performance whereas, in the “beach” and “shore” datasets, the temporal information was not as effective. This is because the “beach” and “shore” datasets are highly dynamic due to moving waves, thus the detector could become overly sensitive to random motions of the waves and cause false positives.

Detecting swimming people in the “beach” dataset is challenging because swimmers only appear as small white dots. The conventional detection methods such as thresholding or background subtraction do not work well due to the safety buoys and moving waves that reflect the background light and also appear as small white dots in the IR images. As shown in Figure 8, these confounding factors can cause multiple false positives for simpler detection methods. On the other hand, the proposed CNN approach is able to take into account higher-level features such as shape and intensity variations that help to distinguish swimmers. Table 4 and Table 5 demonstrate that our proposed method outperforms simpler methods such as background subtraction in the task of human detection.

When compared to the baseline CNN approach, our proposed approach is still able to outperform it in all three datasets according to the results in Table 2, Table 3, Table 4, Table 5, Table 6 and Table 7. The proposed approach has similar object-level detection scores but much higher pixel-level detection scores compared to the baseline CNN. This effect is apparent in Figure 7g and Figure 8g, where the baseline CNN method was only able to identify the coarse outline of people. In contrast, our method was able to label specific pixels accurately. This suggests that our proposed improvements to the CNN training such as weighted loss function and output stride tuning help to improve the network’s ability to capture finer details at the pixel level compared to the baseline CNN.

**Figure 10:**
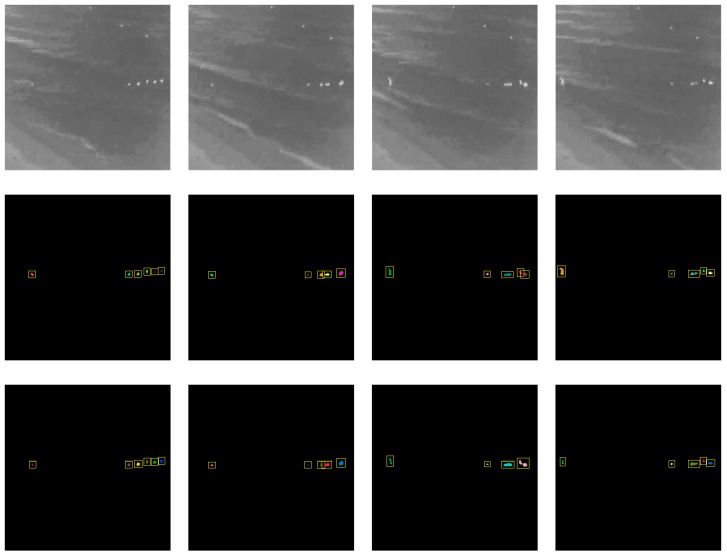
Detection results over time for the beach dataset. The top row shows the original infrared image; the middle row shows the ground truth; the bottom row shows the detection results based on our proposed method.

Figure 10 shows the detection results over time for the beach dataset. The detection results show that the proposed method is able to successfully detect people in the water even though they are constantly moving to different positions in the scene.

**Figure 11:**
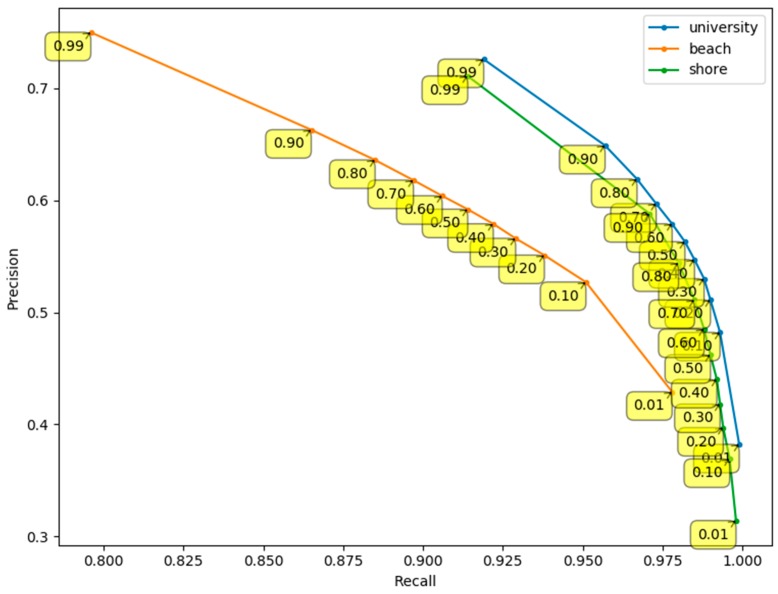
Precision-recall curve of pixel-wise detection results. Yellow boxes indicate the detection threshold at each point on the curve.

Figure 11 shows the precision-recall curve for pixel-wise detections in the “beach”, “shore”, and “university” datasets. The curve is constructed by applying different detection thresholds to the pixel-wise CNN confidence output. As the detection threshold increases, the precision values increase whereas the recall values decrease. To maximize the F1-score, a final detection threshold of 0.99 was used.

To determine whether the detector trained on detecting humans in one dataset is able to generalize and detect humans in another dataset, this research conducted a cross-comparison study where the detector was trained and tested on different datasets. This is a challenging task since the appearance, object sizes, and background scenery between the university and beach datasets are significantly different and some of the features learned might not generalize. Table 8 shows the pixel-level and object-level F1 scores with different train/test configurations. The F1 scores are lower when using a test dataset that is different from the training dataset than when they are the same; however, the results still outperform most of the baseline methods (refer to Table 2, Table 3, Table 4 and Table 5). This suggests that the human detector has some ability to generalize across datasets, even though the objects in the training and test sets are dissimilar. The use of temporal information as CNN input has inconsistent results, i.e., the performance improves under certain configurations but not others. This suggests that the network is sensitive to temporal changes and may overfit to training data with noisy movements in the background.

Table 9 shows the computation time per frame for each method, measured with an Intel Xeon E3-1246 (3.5 GHz) CPU and an NVIDIA GTX 1080 GPU. In general, our method requires more computation time compared to the baseline methods, other than K-means clustering. This is due to the high computational demands of using a deep neural network. However, our method is still able to process the images at a high frame rate (i.e., 6–11 fps), which is sufficient for timely safety warning.

## 4. Conclusions

This study aimed to use infrared cameras to automatically detect people intruding into hazardous areas during night-time to help prevent accidents. To solve this problem, three infrared image datasets for training and testing a person detection algorithm were created. Next, this research developed a CNN-based human detection approach that can perform pixel-wise segmentation and make fine-grained predictions in terms of the object neighborhood. To verify the detection performance of the proposed method, comparative analyses were conducted with four baseline object detection methods. Experimental results demonstrate that the proposed CNN-based human detection approach outperforms conventional detection approaches in all datasets. Especially, the proposed method maintained F1 scores of above 90% in object-level detection for all datasets. Moreover, the computational speed of the proposed method was adequate for real-time safety warning purposes. 

This study is expected to contribute to the safety and security of public areas during night-time by improving the performance of human detection from infrared images. However, since the datasets were obtained at specific times and locations, the proposed method may lack generality when applied to different datasets. Therefore, future research will be focused on constructing datasets with infrared images acquired from a wider variety of viewing angles and locations. In addition, more advanced methods for incorporating temporal image information such as Recurrent Neural Networks (RNN) will be explored.

## Figures and Tables

**Table 1:** Details of each dataset.

Dataset	University	Beach	Shore
Number of images	120	160	540
Resolution	1364 × 1024	1920 × 1080	1920 × 1080
Duration	120 s	40 s	180 s
Sampling rate	1 Hz	4 Hz	3 Hz

**Table 2:** Pixel-level detection score for the university dataset.

Method	Precision	Recall	F1-Score
Simple thresholding	0.040	0.871	0.076
Adaptive thresholding	0.083	0.312	0.131
Background subtraction	0.534	0.951	0.684
K-means clustering	0.037	0.966	0.071
Baseline CNN	0.503	0.901	0.646
Our method	0.726	0.919	0.811
Our method*	0.759	0.948	0.843

(*) indicates that temporal input is used.

**Table 3:** Object-level detection score for the university dataset.

Method	Precision	Recall	F1-Score
Simple thresholding	0.084	0.909	0.153
Adaptive thresholding	0.068	0.932	0.128
Background subtraction	0.506	1.000	0.672
K-means clustering	0.066	0.886	0.122
Baseline CNN	0.976	0.909	0.941
Our method	1.000	0.909	0.952
Our method*	1.000	0.932	0.965

(*) indicates that temporal input is used.

**Table 4:** Pixel-level detection score for the beach dataset.

Method	Precision	Recall	F1-Score
Simple thresholding	0.013	0.971	0.025
Adaptive thresholding	0.802	0.601	0.687
Background subtraction	0.214	0.590	0.314
K-means clustering	0.687	0.725	0.705
Baseline CNN	0.510	0.471	0.490
Our method	0.750	0.796	0.772
Our method*	0.645	0.760	0.698

(*) indicates that temporal input is used.

**Table 5:** Object-level detection score for the beach dataset.

Method	Precision	Recall	F1-Score
Simple thresholding	0.131	0.953	0.230
Adaptive thresholding	0.769	0.836	0.801
Background subtraction	0.313	0.661	0.425
K-means clustering	0.622	0.684	0.652
Baseline CNN	0.971	0.585	0.730
Our method	1.000	0.877	0.935
Our method*	0.961	0.860	0.907

(*) indicates that temporal input is used.

**Table 6:** Pixel-level detection score for the shore dataset.

Method	Precision	Recall	F1-Score
Simple thresholding	0.383	0.801	0.518
Adaptive thresholding	0.254	0.320	0.283
Background subtraction	0.293	0.551	0.383
K-means clustering	0.302	0.649	0.412
Baseline CNN	0.478	0.569	0.520
Our method	0.711	0.914	0.800
Our method*	0.652	0.933	0.768

(*) indicates that temporal input is used.

**Table 7:** Object-level detection score for the shore dataset.

Method	Precision	Recall	F1-Score
Simple thresholding	0.459	0.834	0.593
Adaptive thresholding	0.425	0.788	0.552
Background subtraction	0.269	0.532	0.357
K-means clustering	0.349	0.819	0.490
Baseline CNN	0.974	0.569	0.718
Our method	0.977	0.813	0.888
Our method*	0.948	0.741	0.832

(*) indicates that temporal input is used.

**Table 8:** Cross-comparison of F1-scores with different train/test configurations. Each cell shows the pixel-level F1 score on the left and the object-level F1-score on the right.

	Test Dataset	University	Beach
Train Dataset			
University		0.811 / 0.952	0.436 / 0.749
University*		0.843 / 0.965	0.278 / 0.439
Beach		0.294 / 0.383	0.772 / 0.935
Beach*		0.089 / 0.354	0.698 / 0.907
University + Beach		0.779 / 0.901	0.435 / 0.702
University + Beach*		0.618 / 0.782	0.604 / 0.825

(*) indicates that temporal input is used.

**Table 9:** Computation time per frame for each method.

Method	Dataset #1 Processing Time (s)	Dataset #2 Processing Time (s)	Dataset #3 Processing Time (s)
Simple thresholding	0.001	<0.001	<0.001
Adaptive thresholding	0.008	<0.001	0.003
Background subtraction	0.007	0.001	0.007
K-means clustering	1.947	0.327	2.36
Baseline CNN	0.148	0.102	0.068
Our method	0.126	0.084	0.061
Our method*	0.156	0.088	0.070

(*) indicates that temporal input is used.
